# Preoperative anthropometric assessment of nasal aesthetic parameters in iranian rhinoplasty candidates: deviation from conventional normative standards

**DOI:** 10.1186/s40902-026-00528-6

**Published:** 2026-10-08

**Authors:** Ata Aharipour, Maryam Behdarvandi, Saeed Shirafkan

**Affiliations:** Department of Oral and Maxillofacial Surgery, School of Dentistry, Ahvaz Jundishapur University of Medical Sciences, Ahvaz, Iran

**Keywords:** Rhinoplasty, Esthetic, Photogrammetry, Anthropometry, Nose

## Abstract

**Background:**

Conventional nasal anthropometric norms are widely used in aesthetic analysis; however, their applicability across different populations remains uncertain. This study aimed to perform a preoperative anthropometric assessment of nasal aesthetic parameters in rhinoplasty candidates and to describe the frequency and pattern of deviations from conventional normative criteria.

**Methods:**

In this retrospective descriptive cross-sectional study, preoperative facial photographs of 60 rhinoplasty candidates from private oral and maxillofacial surgery clinics and Imam Khomeini Hospital in Ahvaz, Iran, were evaluated. Preoperative profile and frontal facial photographs meeting predefined quality and positioning criteria were analyzed. Nasal aesthetic parameters were classified as within or outside predefined conventional criteria. Findings were summarized using frequencies, percentages, and exact 95% binomial confidence intervals for the proportions of findings outside the conventional criteria.

**Results:**

The study included 60 rhinoplasty candidates, comprising 32 males (53.3%) and 28 females (46.7%). The most frequent findings outside the conventional criteria were anterior–posterior radix position in 57 patients (95.0%), tip projection in 56 (93.3%), nasofrontal angle in 55 (91.7%), dorsal hump in 54 (90.0%), and alar base width in 47 (78.3%). Vertical radix position, nasolabial angle, and columellar show had relatively balanced distributions within and outside the conventional criteria. The supratip/infratip break criterion was not met in 8 patients (13.3%), and nasal dorsal deviation was present in 11 patients (18.3%).

**Conclusion:**

The findings support the use of anthropometric assessment as a descriptive component of surgical planning, while emphasizing that conventional standards should not be used as the sole determinants of aesthetic suitability. Preoperative evaluation should integrate objective nasal assessment with each patient’s individual goals and cultural preferences.

## Introduction

Rhinoplasty is one of the most frequently performed aesthetic and functional procedures in facial surgery. Because the nose occupies a central position on the face, even subtle variations in nasal morphology can substantially influence the perception of facial attractiveness, balance, and harmony [1, 2]. Accordingly, preoperative nasal assessment commonly relies on anthropometric and photogrammetric analyses, including parameters such as the nasofrontal angle, nasolabial angle, nasal tip projection, alar base width, radix position, dorsal contour, columellar show, and frontal nasal symmetry [3]. These objective measurements allow surgeons to move beyond purely subjective aesthetic judgment and provide a more structured, reproducible, and individualized basis for surgical planning [3].

Although classical and neoclassical standards have long been used in nasal aesthetic analysis, contemporary evidence increasingly suggests that nasal beauty is not a fixed or universal construct [4]. Aesthetic preferences for nasal shape are influenced by ethnicity, culture, sex, age, social background, and evolving beauty trends [5]. Therefore, direct application of generalized or Eurocentric normative standards may be inappropriate for all populations and may fail to account for ethnic and population-specific facial characteristics [4]. In this context, population-sensitive assessment is particularly important in rhinoplasty, where excessive reliance on non-specific ideals may lead to unrealistic surgical goals, overcorrection, or loss of facial identity [5].

In recent years, several studies have emphasized the importance of population-specific nasal analysis. A Thai population-based study reported that preferred nasal profiles differed from some neoclassical facial proportions, including Canon V [6]. Similarly, a Syrian cross-sectional study showed that nasal anthropometric measurements and aesthetic preferences may have distinct population-specific patterns [3]. In Iran, Tavakoli et al. (2023), using three-dimensional imaging, demonstrated that ideal nasal preferences in the Iranian population may differ from some neoclassical standards [7]. These findings support the concept that nasal aesthetic evaluation should not be based solely on universalized norms, but should also consider the anatomical and cultural context of the target population.

Recent three-dimensional computed tomography (CT) studies have further expanded population-specific nasal anthropometry. In a large cohort of 1,022 Turkish rhinoplasty candidates, Tuncel et al. demonstrated significant differences in the dorsal profile, nasofrontal, and kyphion angles between S- and V-shaped nasal bones and highlighted the clinical relevance of the dorsal profile angle [8]. More recently, Kokten et al. reported positive correlations between nasal height and horizontal facial and nasal dimensions in 249 Turkish rhinoplasty candidates, together with notable sex-related differences. These findings demonstrate the value of three-dimensional, population-specific assessment of nasal morphology in rhinoplasty planning [9].

However, an important distinction should be made between aesthetic preference studies, normative measurements in the general population, and the actual preoperative morphology of patients seeking rhinoplasty [10]. Preference-based studies indicate what individuals may perceive as aesthetically desirable, whereas clinical preoperative assessment reflects the anatomical patterns that surgeons encounter in real surgical candidates [6, 11]. Patients presenting for rhinoplasty may show concurrent deviations across several nasal parameters rather than isolated abnormalities [12]. Therefore, evaluating single measurements or idealized nasal profiles may provide an incomplete representation of the clinical challenges involved in surgical decision-making [13].

This issue is especially relevant in Iranian rhinoplasty candidates, given the high demand for rhinoplasty in Iran and the potential mismatch between conventional aesthetic standards and Iranian nasal-facial morphology [5, 14]. While previous Iranian studies have examined selected nasal measurements in the general population [15, 16] or explored subjective aesthetic preferences using digitally manipulated or three-dimensional images [7], evidence remains limited regarding the objective and simultaneous assessment of multiple profile- and frontal-view nasal parameters in a clinical population actively seeking rhinoplasty [17]. Because nasal aesthetics are determined by the interaction of profile and frontal features, combined evaluation of these parameters may better reflect real preoperative morphology and provide more clinically meaningful information than isolated measurements alone [18].

Therefore, a comprehensive preoperative anthropometric assessment of Iranian rhinoplasty candidates may help identify which nasal aesthetic parameters most frequently deviate from conventional normative standards [16]. Such information may support more realistic preoperative counseling, reduce excessive dependence on non-population-specific ideals, help avoid overcorrection, and contribute to culturally and anatomically sensitive surgical planning [19, 20]. Unlike previous studies focusing primarily on individual nasal measurements, idealized aesthetic preferences, or general-population samples, limited evidence is available on the simultaneous assessment of multiple nasal parameters in a clinical population of Iranian patients actively seeking rhinoplasty. The present study addresses this gap by concurrently evaluating multiple profile- and frontal-view parameters, thereby providing a clinically oriented description of the pattern of coexisting deviations encountered during actual preoperative assessment. Accordingly, the present study aimed to perform a preoperative anthropometric assessment of nasal aesthetic parameters in Iranian rhinoplasty candidates and to determine their pattern of deviation from conventional normative standards.

## Materials and methods

### Study design and setting

This retrospective descriptive cross-sectional study was conducted using preoperative facial photographs available in the patients’ clinical records. The study population consisted of rhinoplasty candidates referred to private oral and maxillofacial surgery clinics and Imam Khomeini Hospital in Ahvaz, Iran, in 2018. The primary objective was to evaluate the pattern of deviation of nasal aesthetic parameters from conventional anthropometric standards among Iranian rhinoplasty candidates.

### Participants and sample size

The study sample included 60 patients referred to private oral and maxillofacial surgery clinics and Imam Khomeini Hospital in Ahvaz. Patients were eligible for inclusion if standardized preoperative photographs were available and if the required nasal aesthetic parameters could be assessed in both profile and frontal views. Patients were excluded if they had a history of previous rhinoplasty or other nasal surgery, nasal trauma or fracture, congenital craniofacial anomalies, previous facial surgery or significant facial injury, or any condition affecting nasal morphology. Patients with incomplete or poor-quality photographs, non-standard head positioning, or insufficient visualization of the required facial landmarks were also excluded. The sample size was calculated using the single-proportion formula, as shown in Eq. 1. In this formula, *p* = 0.50 represents the expected prevalence, d = 0.13 represents the desired precision, and Z₁₋α/₂ = 1.96 represents the standard normal value at the 95% confidence level. The minimum required sample size was estimated to be approximately 57 patients; therefore, 60 patients were included in the final analysis.

$$\:n=\frac{{\left({Z}_{1}-\raisebox{1ex}{$\alpha\:$}\!\left/\:\!\raisebox{-1ex}{$2$}\right.\right)}^{2}\:p\:(1-p)}{{\left(d\right)}^{2}}\:=\:\frac{{\left(1.96\right)}^{2}\:0.5\left(1-0.5\right)}{{\left(0.13\right)}^{2}}=56.8\:\approx\:57\:\:\:\:\left(1\right)$$

### Photographic assessment

Routine preoperative facial photographs available in the patients’ clinical records were retrospectively evaluated. For each participant, frontal and true lateral profile photographs were reviewed. Before measurement, all images were screened for suitability for anthropometric assessment. Photographs were considered eligible when they demonstrated adequate resolution and exposure, complete visualization of the required facial and nasal landmarks, a neutral facial expression, and acceptable frontal and true lateral profile views. For frontal photographs, the face was required to be directed toward the camera without evident rotation or lateral tilting. For lateral photographs, a true profile view without evident head rotation, flexion, or extension was required. Photographs with inadequate exposure or resolution, incomplete visualization of the required landmarks, improper head positioning, a non-neutral facial expression, or obvious perspective distortion were excluded.

#### Image calibration and measurement instruments

Digital photographs were analyzed using ImageJ software, version 1.52 (National Institutes of Health, Bethesda, MD, USA). Before performing linear measurements, each image was calibrated individually. The known actual length of the calibration marker visible in each photograph was entered into ImageJ using the Set Scale function, allowing pixel distances to be converted into millimeters. A calibration marker of known length was present in all photographs used for linear measurements. However, because the photographs were retrospectively obtained from routine clinical records, detailed information regarding the type and exact positioning of the calibration marker and the consistency of its distance from the subject could not be retrospectively verified.

All linear measurements were subsequently obtained in millimeters using the image-specific calibration scale. For angular measurements, the photographs were printed without altering their original aspect ratio. After the relevant anatomical landmarks and reference lines were identified and drawn on the printed photographs, the angles were measured manually with a standard goniometer and protractor. This manual approach reflected the measurement protocol used during the original assessment.

#### Radix assessment

**Fig. 1:**
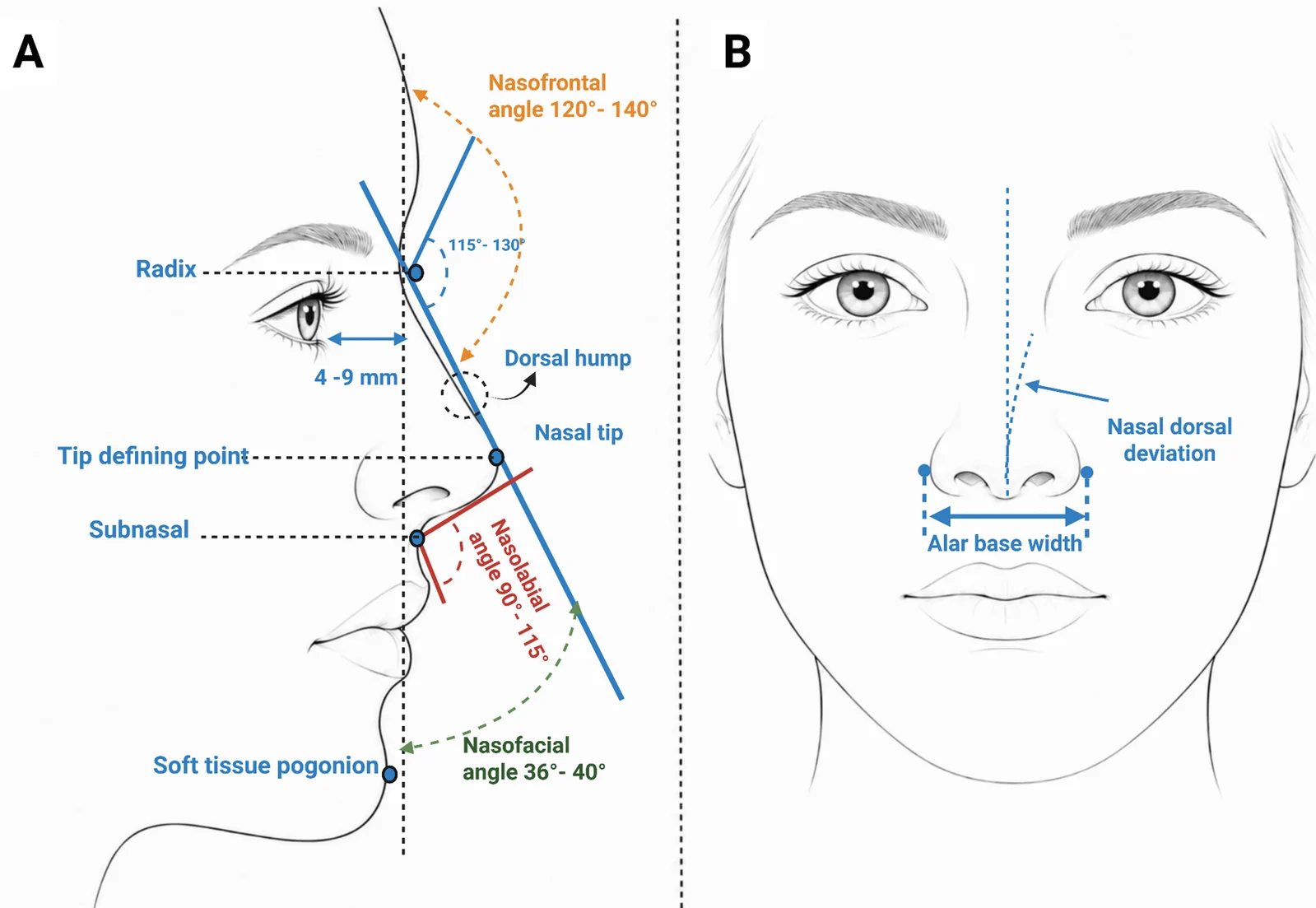
Anthropometric parameters used for nasal aesthetic assessment in profile and frontal views. (**A**) Profile-view parameters, including radix position, dorsal hump, nasal tip projection, soft tissue pogonion, and subnasal position. (**B**) Frontal-view parameters, including nasal dorsal deviation and alar base width

The soft-tissue radix was identified as the deepest point of the frontonasal concavity in the true lateral profile. Radix position was evaluated separately in the anterior–posterior and vertical dimensions; no independent variable termed “radix height” was measured. For the anterior–posterior assessment, the anterior corneal plane was defined as a vertical line tangent to the most anterior surface of the cornea. The shortest perpendicular distance between the soft-tissue radix and this plane was measured on the calibrated digital image using ImageJ. The vertical radix position was assessed by comparing the level of the soft-tissue radix with the upper-eyelid lash line and supratarsal crease. The anatomical reference points used for radix assessment are illustrated in Fig. 1A, and the criteria used to classify the anterior–posterior and vertical radix positions as within or outside the conventional reference criteria are presented in Table 1.

#### Profile-view measurements

The nasofrontal angle was measured at the soft-tissue radix between a line tangent to the forehead and a line tangent to the nasal dorsum. The nasolabial angle was measured at the subnasale between the columellar line and the upper-lip line. Dorsal contour was evaluated relative to a straight line connecting the radix and nasal tip. Tip projection was assessed using Goode’s ratio, calculated as tip projection divided by nasal length. Columellar show and the presence or absence of appropriate supratip and infratip breaks were evaluated in the true lateral profile (Fig. 1A; Table 1).

#### Frontal-view assessment

Alar base width was measured on the calibrated frontal image as the distance between the right and left alare points and was compared with the intercanthal distance. The facial midline was defined as a vertical line extending from the midglabellar point to the soft-tissue menton. The presence or absence of visible deviation of the external nasal dorsum from this line was assessed qualitatively. Nasal dorsal deviation was recorded as a binary variable, and its angular or linear magnitude was not quantified (Fig. 1B; Table 2).

**Table 1:** Operational definitions and conventional reference criteria for profile-view nasal aesthetic parameters

Parameter	Operational definition/conventional reference criterion
Anterior–posterior radix position	The anterior–posterior radix position was considered within the conventional reference criterion when located approximately 4–9 mm anterior to the corneal plane [21, 22].
Vertical radix position	The vertical radix position was assessed in relation to the superior palpebral margin and upper eyelid margin [21].
Nasofrontal angle	The conventional reference range for the nasofrontal angle was defined as 115°–130° [21].
Dorsal hump	In women, the nasal dorsum was considered ideal when approximately 2 mm below a line connecting the radix to the nasal tip; in men, the dorsum was expected to lie at or slightly below this line [23].
Supratip and infratip break	The presence or absence of appropriate supratip and infratip definition was recorded [20].
Tip projection	Nasal tip projection was evaluated using Goode’s analysis, with a conventional reference ratio of approximately 0.55–0.60 [24].
Columellar show	The degree of visible columella was evaluated in the profile view [20].
Nasolabial angle	The conventional reference range was defined as 95°–110° in women and 90°–95° in men [24].

**Table 2:** Operational definitions and conventional reference criteria for frontal-view nasal aesthetic parameters

Parameter	Operational definition/conventional reference criterion
Alar base width	Alar base width was considered within the conventional reference criterion when approximately equal to the intercanthal distance [18].
Nasal dorsal deviation	The presence or absence of visible deviation of the external nasal dorsum from the facial midline was assessed in the frontal view [25].

#### Measurement reliability

The photographs were independently evaluated by two assessors using the same operational definitions and classification criteria. Interobserver agreement was determined by comparing the classifications made by the two assessors. For intraobserver reliability, each assessor repeated the evaluations in a separate session. Interobserver and intraobserver agreement for the binary classifications was assessed using Cohen’s kappa coefficient.

### Outcome definition

For each parameter, findings were classified as either within or outside the predefined conventional reference criteria. The primary outcome was the frequency and percentage of participants whose measurements or findings were outside the conventional reference criteria for each nasal aesthetic parameter. The two classification categories were analyzed separately for each parameter. At the time of the original data recording, only the final binary classification of each parameter as within or outside the predefined conventional reference criteria was retained in the study dataset, whereas the underlying continuous measurement values were not retained. Consequently, these continuous values were unavailable for the present retrospective analysis, preventing assessment of the magnitude and direction of deviations from the reference criteria.

### Statistical analysis

Data were analyzed using Minitab Statistical Software, version 21. All nasal aesthetic parameters were analyzed as binary categorical variables and classified as either within or outside the predefined conventional reference criteria. Frequencies and percentages were used to summarize the overall and sex-specific distributions of these two classification categories. Two-sided 95% confidence intervals for the proportions of findings outside the conventional reference criteria were calculated using the exact binomial method. Normality testing was not applicable because the available variables were binary categorical data. No inferential hypothesis testing was performed because no prespecified or theoretically justified expected distribution of findings within and outside the conventional reference criteria was available.

## Results

### Measurement reliability

Interobserver kappa values ranged from 0.73 to 0.86 across the evaluated parameters. Intraobserver kappa values ranged from 0.79 to 0.89 for the first assessor and from 0.81 to 0.88 for the second assessor, indicating substantial to almost perfect agreement.

### Distribution of nasal aesthetic findings

Complete data were available for all 60 rhinoplasty candidates, including 28 females and 32 males. The overall and sex-specific distributions of findings within and outside the conventional reference criteria, together with exact 95% confidence intervals for the proportions outside these criteria, are presented in Table 3.

**Table 3:** Distribution of findings within and outside the conventional reference criteria

View	Parameter	Outside reference criteria, *n* (%)	Within reference criteria, *n* (%)	Female (Outside/Within)	Male (Outside/Within)	95% CI for proportion outside reference criteria
Profile	Anterior–posterior radix position	57 (95)	3 (5)	26/2	31/1	86.1–99.0%
	Vertical radix position	32 (53.3)	28 (46.7)	16/12	16/16	40.0–66.3%
	Nasofrontal angle	55 (91.7)	5 (8.3)	26/2	29/3	81.6–97.2%
	Dorsal hump	54 (90)	6 (10)	26/2	28/4	79.5–96.2%
	Supratip and infratip break	8 (13.3)	52 (86.7)	6/22	2/30	5.9–24.6%
	Tip projection	56 (93.3)	4 (6.7)	27/1	29/3	83.8–98.2%
	Columellar show	27 (45)	33 (55)	9/19	18/14	32.1–58.4%
	Nasolabial angle	31 (51.7)	29 (48.3)	14/14	17/15	38.4–64.8%
Frontal	Alar base width	47 (78.3)	13 (21.7)	21/7	26/6	65.8–87.9%
	Nasal dorsal deviation	11 (18.3)	49 (81.7)	7/21	4/28	9.5–30.4%

The 95% confidence intervals were calculated for the proportions of findings outside the conventional reference criteria using the exact binomial method

**Fig. 2:**
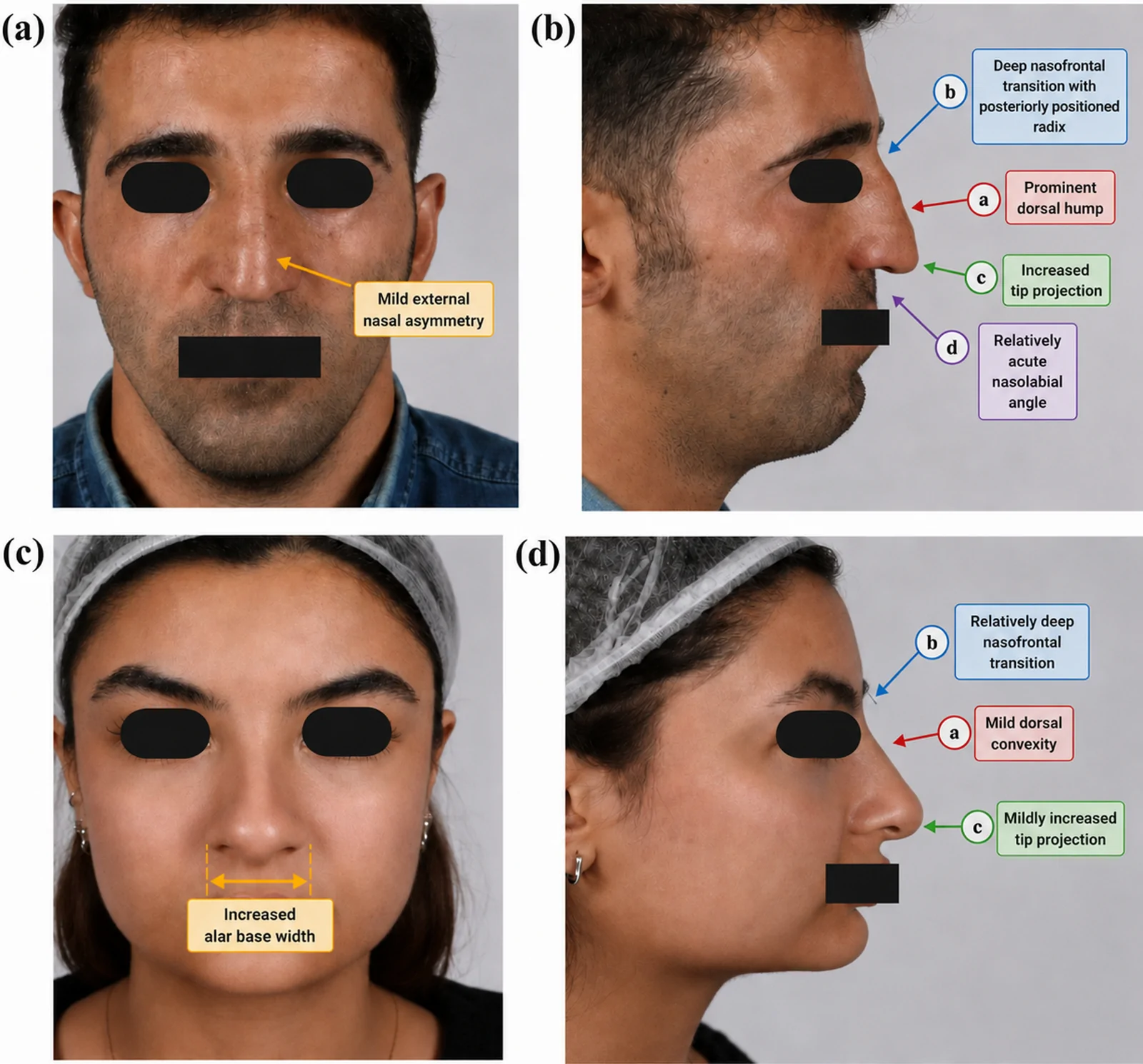
Representative preoperative clinical photographs illustrating nasal features observed in rhinoplasty candidates. (**a**) Frontal view demonstrating mild external nasal asymmetry. (**b**) Lateral view showing a prominent dorsal hump, a deep nasofrontal transition with a posteriorly positioned radix, increased tip projection, and a relatively acute nasolabial angle. (**c**) Frontal view demonstrating increased alar base width. (**d**) Lateral view showing mild dorsal convexity, a relatively deep nasofrontal transition, and mildly increased tip projection

Representative preoperative frontal and lateral clinical photographs illustrating the evaluated nasal features are presented in Fig. 2.

In the profile view, the highest proportions outside the conventional reference criteria were observed for anterior–posterior radix position, tip projection, nasofrontal angle, and dorsal contour. Specifically, anterior–posterior radix position was outside the reference criterion in 57 participants (95.0%), including 26 females (92.9%) and 31 males (96.9%). Tip projection was outside the reference range in 56 participants (93.3%), comprising 27 females (96.4%) and 29 males (90.6%). The nasofrontal angle was outside the reference range in 55 participants (91.7%), including 26 females (92.9%) and 29 males (90.6%). Dorsal contour was outside the reference criterion in 54 participants (90.0%), comprising 26 females (92.9%) and 28 males (87.5%).

By contrast, vertical radix position, nasolabial angle, and columellar show had relatively balanced distributions between the two classification categories. Vertical radix position was outside the reference criterion in 32 participants (53.3%), including 16 females (57.1%) and 16 males (50.0%). The nasolabial angle was outside the reference range in 31 participants (51.7%), comprising 14 females (50.0%) and 17 males (53.1%). Columellar show was outside the reference criterion in 27 participants (45.0%), including 9 females (32.1%) and 18 males (56.3%).

The conventional supratip/infratip break criterion was met in 52 participants (86.7%) and was not met in 8 participants (13.3%), including 6 females and 2 males. In the frontal view, alar base width was outside the conventional reference criterion in 47 participants (78.3%), including 21 females (75.0%) and 26 males (81.3%). Visible nasal dorsal deviation was absent in 49 participants (81.7%), comprising 21 females (75.0%) and 28 males (87.5%), and was present in 11 participants (18.3%), including 7 females (25.0%) and 4 males (12.5%). Overall, the highest proportions outside the conventional reference criteria were observed for anterior–posterior radix position, tip projection, nasofrontal angle, dorsal contour, and alar base width.

## Discussion

In the present study, a comprehensive evaluation of nasal anthropometric parameters and facial angles was performed in Iranian rhinoplasty candidates using predefined conventional reference criteria. These descriptive findings illustrate the value of assessing multiple nasal features during preoperative evaluation. However, findings outside conventional reference criteria should not necessarily be interpreted as undesirable aesthetic features or as inconsistent with patients’ individual.

In the present study, the nasofrontal angle was classified as outside the conventional reference range in most participants, whereas the nasolabial angle showed a relatively balanced distribution between findings within and outside the conventional reference range. The clinical relevance of these angular parameters has also been demonstrated in studies of postoperative nasal morphology. For instance, Zojaji et al. [26] reported postoperative increases in the nasofrontal angle from 130° to 132.9°, the nasolabial angle from 93.6° to 98.9°, and the nasomental angle from 126.6° to 128.1°. These changes may result from modification of the osteocartilaginous framework and controlled rotation of the nasal tip, which alter the angular relationships between the nose and adjacent facial structures [27, 28]. In particular, an increase in the nasolabial angle may reflect cephalic tip rotation and altered nasolabial harmony [29]. Although these postoperative changes are not directly comparable with the preoperative classifications used in the present study, they demonstrate the clinical relevance of angular assessment in rhinoplasty.

Among the linear and proportional parameters evaluated in the present study, tip projection and alar base width were classified as outside the conventional range in 93.3% and 78.3% of participants, respectively. The clinical relevance of these parameters has also been demonstrated in studies assessing postoperative changes. In this context, Zojaji et al. [26] reported postoperative reductions in nasal length from 4.9 to 4.8 cm, alar base width from 3.4 to 3.2 cm, and tip projection from 3.3 to 3.1 cm. In addition, Wang et al. noted that the proportional relationships among nasal length, tip projection, dorsal height, and radix height were 2:0.97:0.61:0.28, respectively, in young Korean individuals. They also observed lower tip projection and dorsal height in individuals with saddle nose deformity [30]. Although these studies differ from the present study in population, design, and measurement approach, they demonstrate the clinical importance of evaluating nasal projection and width in relation to the overall nasal framework.

Moreover, tip projection was frequently classified as outside the conventional range in the present study, whereas visible nasal dorsal deviation was identified in a smaller proportion of participants. In this context, Choi et al. demonstrated that the nasolabial and nasomental angles influence the perceived ideal position of the nasal tip, while other variables, including the midface-to-lower-face ratio, had no significant effect [31]. From a postoperative perspective, Yi et al. [32], reported that 71% of patients undergoing septoplasty with caudal septal batten grafting had preoperative nasal deformities and that dorsal deviation improved in 61% following surgery. However, most anthropometric parameters, including the nasolabial and nasofrontal angles, remained unchanged. Although these postoperative findings are not directly comparable with the descriptive preoperative findings of the present study, they indicate that individual nasal features may respond differently according to the objectives and techniques of surgery. In particular, batten grafting primarily addresses structural support and alignment rather than comprehensive aesthetic modification [33].

Population-specific differences also play a critical role. Tavakoli et al. [7], in a sample of 203 Iranian participants, reported preferred nasofrontal angles of 137.64° ± 4.20° in males and 133.55° ± 4.53° in females, which are notably higher than classical neoclassical values. The preferred nasolabial angles were 98.92° ± 4.88° in males and 107.56° ± 5.20° in females, while ideal tip projection ratios were approximately 0.56–0.57. These findings suggest that classifications based exclusively on conventional reference ranges may not fully reflect population-specific aesthetic preferences. Accordingly, the high frequency of findings outside these ranges in the present study should not necessarily be interpreted as indicating undesirable nasal morphology. Similarly, Mulafikh et al. [34] reported mean nasofacial, nasofrontal, and nasolabial angles of 29.7° ± 3.4°, 146.2° ± 2.8°, and 113.5° ± 11°, respectively, in Arabian women seeking rhinoplasty. These values indicate a tendency toward a more obtuse nasofrontal angle and relatively moderate tip rotation, which supports the interpretation that aesthetic norms vary considerably across populations.

Recent three-dimensional CT studies further demonstrate the importance of population- and sex-specific nasal assessment. Tuncel et al. reported significant differences in the dorsal profile, nasofrontal, and kyphion angles between S- and V-shaped nasal bones and highlighted the clinical relevance of the dorsal profile angle [8]. Similarly, Kokten et al. found positive correlations between nasal height and horizontal facial and nasal dimensions, with more pronounced relationships in males. Although these studies primarily evaluated the bony nasal framework, their findings support interpreting nasal parameters as interrelated and population-specific characteristics rather than isolated universal measurements [9].

Another critical methodological consideration is the effect of body position on nasal measurements. Kim et al. demonstrated that the nasofrontal angle was significantly greater in the supine position compared to the erect position, while other parameters such as the nasolabial and nasomental angles remained unchanged [35]. This perspective supports interpreting the deviations from conventional reference ranges observed in the present study within a population-specific context rather than as definitive aesthetic abnormalities. Finally, Doddi et al. [36], in a systematic review, emphasized that although anthropometric data provide objective measurements, substantial overlap exists among populations, thereby limiting their utility in defining universal aesthetic standards. This perspective supports interpreting the deviations from conventional reference ranges observed in the present study within a population-specific context rather than as definitive aesthetic abnormalities [36].

From a clinical perspective, these findings suggest that conventional anthropometric criteria may be most useful as reference points rather than fixed surgical targets. During preoperative planning, surgeons may benefit from evaluating multiple nasal parameters together and identifying which features classified as outside conventional criteria are actually relevant to the patient’s concerns and desired outcome. A finding outside a conventional range should therefore not automatically prompt surgical correction. Instead, such findings may serve as a basis for a more structured discussion with patients regarding their individual nasal morphology, expectations, and the potential trade-offs of modifying specific features. This approach may help avoid overcorrection and support more individualized and culturally sensitive surgical planning.

However, these clinical implications should be interpreted in light of several methodological considerations. A further methodological consideration is the use of routine clinical photographs that were not acquired under a fully standardized photographic protocol. Variability in camera specifications, camera-to-subject distance, lighting conditions, head positioning, and landmark visualization may have affected measurement accuracy and introduced additional measurement variability. In addition, angular measurements were performed manually on printed photographs using a goniometer and protractor rather than digitally, which may have introduced observer-dependent measurement error. Although the observed interobserver and intraobserver agreement was substantial to almost perfect, these methodological factors should be considered when interpreting the anthropometric findings.

## Limitations

The present study did not directly assess patients’ aesthetic concerns, cultural preferences, or expectations regarding rhinoplasty. Moreover, the available dataset contained only binary classifications indicating whether each finding was within or outside the conventional reference criteria, preventing assessment of the magnitude and direction of deviations. Therefore, findings outside the conventional reference criteria should not necessarily be interpreted as features considered undesirable by patients. Future studies should record continuous measurements and combine anthropometric assessment with culturally sensitive evaluations of patients’ preferences and desired outcomes. In addition, although the sample size was adequate for the overall descriptive objective of the study, its relatively small size limits the precision of the estimates, particularly for parameters with lower observed frequencies, as well as the reliability of subgroup comparisons and the generalizability of the findings to the broader population of Iranian rhinoplasty candidates. Because the photographs were obtained as part of routine clinical documentation, complete information regarding camera specifications, lighting conditions, camera-to-subject distance, and photographer-related variability could not be retrospectively verified. Similarly, because this was a retrospective study, the original rationale for measuring angular parameters manually with a goniometer/protractor rather than digitally in ImageJ was not documented and could not be reconstructed.

## Conclusion

The observed pattern of nasal features in this cohort suggests that conventional anthropometric criteria alone may not adequately characterize the preoperative morphology of Iranian rhinoplasty candidates. Anthropometric assessment can provide a structured basis for surgical planning; however, its findings should be interpreted in conjunction with the patient’s individual aesthetic goals and cultural preferences. Future studies using larger and more diverse samples, continuous measurements, and direct assessments of patient preferences are needed to develop more population-sensitive approaches to preoperative nasal evaluation.

## Data Availability

No datasets were generated or analysed during the current study.
